# Severe postoperative infection after intramuscular silicone buttock augmentation via intergluteal fold incision: Case report and management

**DOI:** 10.1016/j.jpra.2025.09.011

**Published:** 2025-09-12

**Authors:** Ingrid C. Landfald

**Affiliations:** a Department of Clinical Anatomy, Mazovian Academy in Płock, Płock, Poland; b VARIANTIS Research Laboratory, Department of Clinical Anatomy, Mazovian Academy in Płock, Płock, Poland

**Keywords:** Buttock augmentation, Silicone implants, Intramuscular placement, Surgical infection, Staphylococcusinfection, Wound management

## Abstract

**Background:**

Intramuscular buttock augmentation with silicone implants can produce immediate and durable contour enhancement but carries notable complication risks, particularly postoperative infection. The intergluteal fold approach, although offering optimal scar concealment, presents anatomical vulnerabilities that may predispose to contamination and impaired wound healing.

**Case Presentation:**

A 21-year-old woman developed a severe postoperative infection following bilateral intramuscular buttock augmentation via a 7 cm intergluteal fold incision. On postoperative day 5, she presented with severe gluteal pain, swelling, erythema, and systemic symptoms. Computed tomography demonstrated bilateral periprosthetic fluid collections and subcutaneous emphysema. Microbiological culture identified coagulase-negative *Staphylococcus* sensitive to beta-lactams.

**Management and Outcome:**

The patient underwent urgent intravenous antibiotics, targeted antimicrobial adjustment, and staged surgical wound management involving deliberate incision reopening, thorough irrigation, and passive drainage, without implant removal. Complete infection resolution was achieved by postoperative day 21, though notable intergluteal scarring developed.

**Conclusions:**

This case illustrates the infection risk associated with the intergluteal fold approach in intramuscular gluteal augmentation. Early recognition, aggressive multidisciplinary management, and carefully selected implant-preserving strategies may achieve resolution without explantation. Surgeons must weigh aesthetic advantages against anatomical infection risks and ensure comprehensive patient counseling.

## Introduction

Intramuscular buttock augmentation with silicone implants has gained popularity for its ability to deliver immediate, natural-appearing, and durable contour enhancement. Modern gluteal implants are engineered to replicate the viscoelastic properties of native soft tissue, ensuring long-term stability and patient satisfaction.^1^ The intergluteal fold incision is widely preferred as it provides effective scar concealment and direct access to the intramuscular plane for precise implant placement.^2^

Despite these benefits, the technique carries inherent risks. The intergluteal fold is anatomically close to the anal verge, increasing the likelihood of contamination from the perianal region. In addition, the relatively limited vascular supply in this area may reduce local immune defense and impair wound healing. Reported postoperative infection rates following gluteal implant surgery range from 1.9 % to 7.0 %, with overall complication rates as high as 30 %.^2^^,^^3^

Recent evidence shows that pathogens such as Cutibacterium avidum, not traditionally linked to early surgical site infections, have been identified as causative organisms in buttock implant infections, emphasizing the need for accurate microbiological diagnosis and targeted therapy.^3^ Severe complications have also been reported after gluteal filler procedures, where minimally invasive management with irrigation and passive drainage achieved resolution without implant removal in selected cases.^4^

We present a case of severe postoperative infection following intramuscular silicone buttock augmentation via the intergluteal fold approach, managed successfully with aggressive medical and surgical interventions without explantation. This report underscores the importance of early recognition, tailored management, and consideration of implant preservation in appropriate patients.

## Case report

### Clinical presentation

A previously healthy 21-year-old woman underwent elective bilateral buttock augmentation with 330 mL round, smooth silicone implants. The implants were inserted into the intramuscular plane via a vertical intergluteal fold incision measuring approximately 7 cm in length. Prophylactic oral clindamycin was administered immediately after surgery.

On postoperative day 5, the patient presented to the emergency department with severe left-sided gluteal pain, marked swelling, erythema, and tenderness. She also reported malaise and subjective fever. Laboratory tests revealed elevated inflammatory markers, with C-reactive protein (CRP) at 43 mg/L and a white blood cell count (WBC) of 8.0 × 10⁹/L.

**Figure 1:**
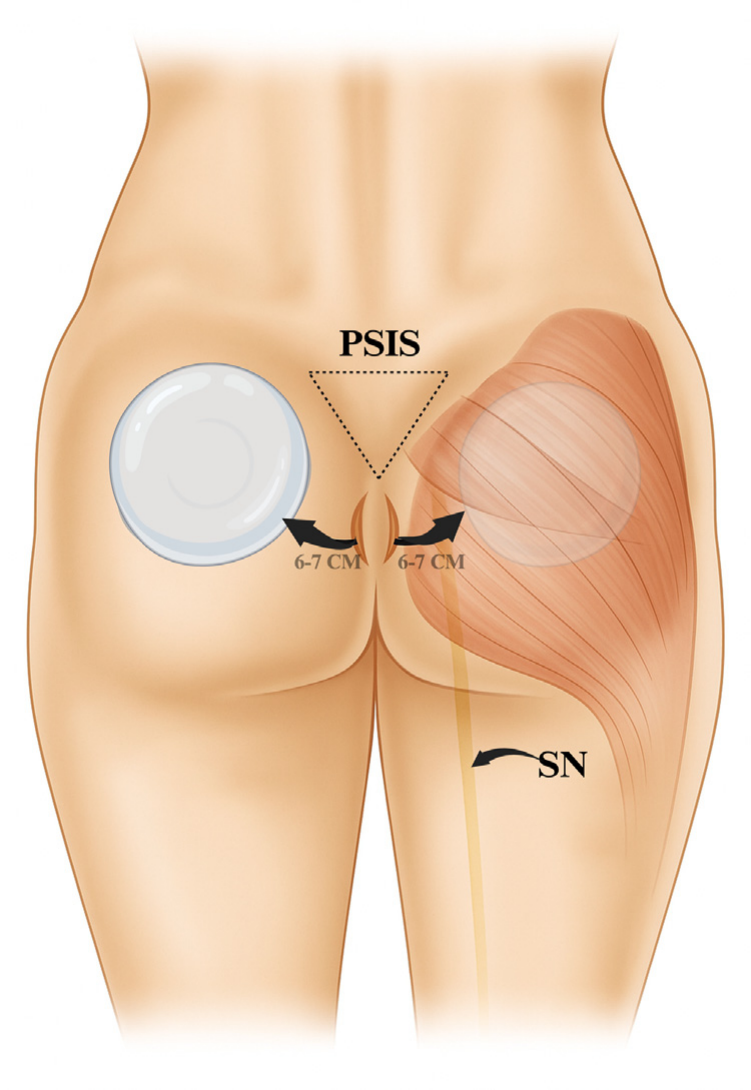
Surgical technique for intramuscular gluteal implant placement. Schematic illustration of bilateral insertion of 330 mL round, smooth silicone implants through 6–7 cm vertical intergluteal fold incisions. The drawing demonstrates creation of the intramuscular pocket within the gluteus maximus, ensuring stable implant positioning and concealed scarring. The course of the sciatic nerve (SN) is indicated to highlight its anatomical relationship and the safety margin preserved during implant placement.

Figure 1 depicts the surgical approach, illustrating implant positioning within the gluteus maximus via the intergluteal fold incision for optimal scar concealment and implant stability.

## Diagnostic assessment

**Figure 2:**
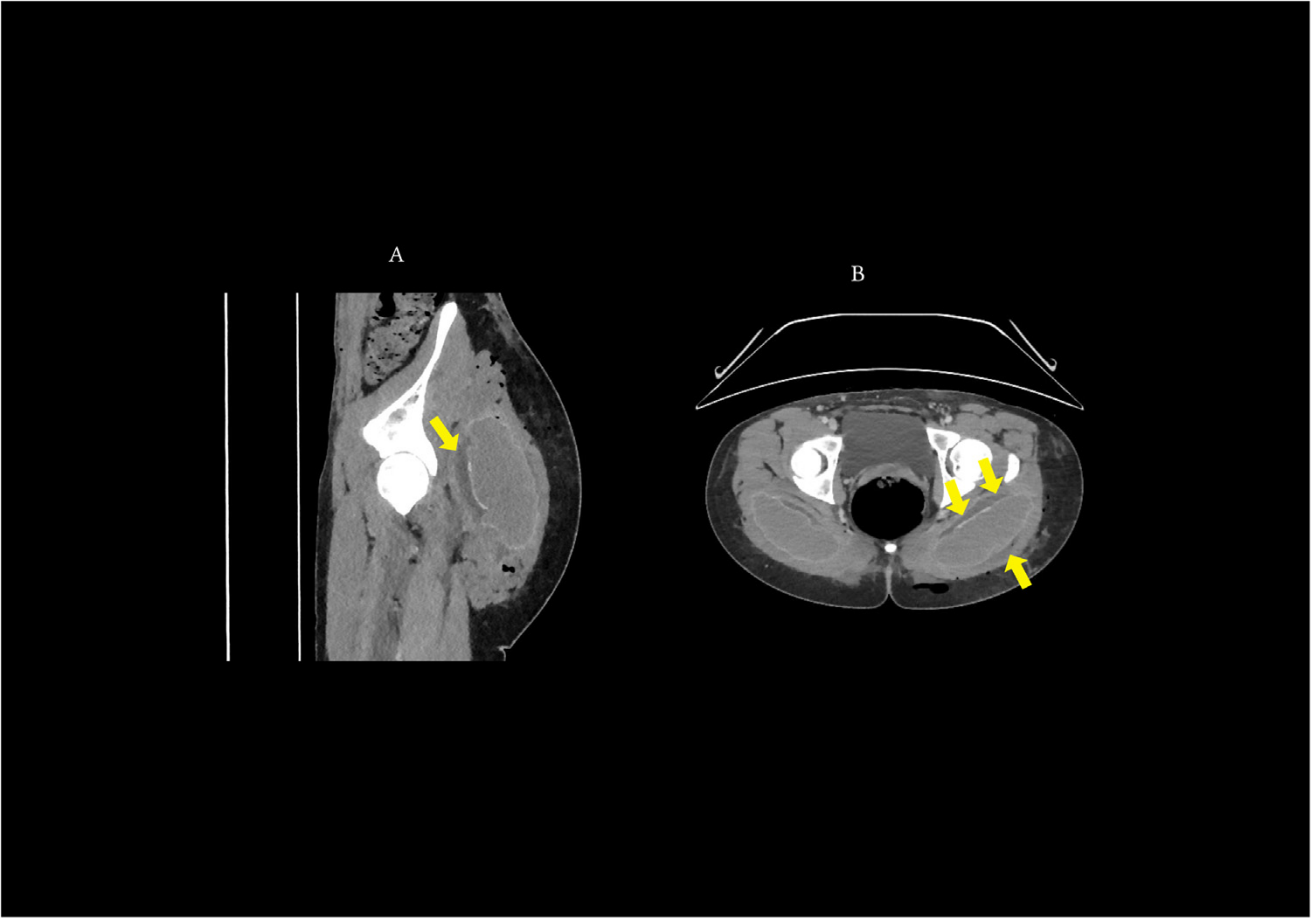
Computed tomography (CT) findings in postoperative gluteal infection. A: Sagittal CT image of the left buttock showing a periprosthetic fluid collection surrounding the silicone implant (yellow arrow) within the intramuscular plane of the gluteus maximus, with adjacent gas locules suggestive of infection. B: Axial CT image demonstrating bilateral periprosthetic fluid collections (yellow arrows) and subcutaneous emphysema, more pronounced on the left side, consistent with severe postoperative infection.

An urgent computed tomography (CT) scan of the pelvis demonstrated bilateral periprosthetic fluid collections within the intramuscular plane, extending along the gluteus maximus and more prominent on the left side. Extensive subcutaneous emphysema was noted, predominantly in the left gluteal and perianal regions, with multiple intramuscular gas locules suggestive of an infectious process with possible necrotizing components (Figure 2).

## Surgical and medical management

The patient was admitted for intravenous broad-spectrum antibiotic therapy with cefotaxime and metronidazole. On postoperative day 7, spontaneous dehiscence of the left intergluteal fold incision occurred, releasing copious purulent material. Wound swab cultures, processed under both aerobic and anaerobic conditions, grew coagulase-negative *Staphylococcus* sensitive to beta-lactams. Based on sensitivity results, antimicrobial therapy was adjusted to oral ampicillin (500 mg three times daily), clindamycin (300 mg four times daily), and ciprofloxacin (500 mg twice daily).

Despite targeted antimicrobial therapy, the patient exhibited persistent local signs of infection. Surgical re-intervention under local anesthesia was undertaken on the contralateral (right) incision, which was reopened, thoroughly irrigated with sterile saline, and deliberately left unsutured to allow passive drainage. The wound edges were loosely approximated with sterile adhesive strips to maintain aeration and facilitate continuous egress of exudate. This approach was chosen to optimize drainage and reduce the risk of reinfection without necessitating implant removal.

## Outcome

By postoperative day 21, clinical and laboratory parameters confirmed complete infection resolution without implant explantation. The patient developed a visible linear scar within the intergluteal fold, which she reported as aesthetically unsatisfactory. At 6-month follow-up, the patient was asymptomatic with complete wound healing and no recurrence of infection. At 12 months, clinical review confirmed stable implants, thin and well-healed bilateral scars, and no late complications.

## Discussion

Intramuscular silicone buttock augmentation via the intergluteal fold approach offers optimal scar concealment and stable aesthetic outcomes. However, this incision site lies in close proximity to the anal verge and within a region of relatively reduced vascularity, predisposing the surgical field to contamination and impaired healing.^1^^,^^2^ Reported infection rates in gluteal implant surgery remain clinically significant, and the sequelae can be severe, often necessitating implant removal.^3^ In the present case, bilateral vertical intergluteal fold incisions were used, a variation that may reduce midline tension but does not mitigate the intrinsic risk of contamination associated with this approach.

Early recognition of infection, prompt imaging, and immediate initiation of intravenous antibiotics were critical in preventing further progression. Instead of implant removal, an implant-sparing strategy with staged wound reopening, irrigation, and passive drainage was employed. The favorable outcome was likely due to the combination of timely intervention, culture-guided antimicrobial therapy, and maintenance of continuous drainage, all of which reduced bacterial load while preserving implant stability. This management parallels recent findings by Kristiansen et al.,^4^ who described successful outcomes with minimally invasive wound approaches in severe gluteal complications.

Pathogen identification remains a crucial step, as organisms beyond *Staphylococcus* spp., notably *Cutibacterium avidum*, have been implicated in gluteal implant infections.^3^ Another important consideration is bacterial biofilm, which can cause delayed or recurrent infection despite apparent resolution. In this case, structured follow-up at 6 and 12 months confirmed complete recovery, stable implant positioning, and thin, well-healed scars, with no evidence of biofilm-related complications or capsular contracture.

Finally, this case underscores the importance of thorough preoperative counseling, which should address both aesthetic expectations and the potential for adverse outcomes such as infection, scarring, or implant loss. Standardized postoperative care pathways with clear protocols for early intervention may improve both patient safety and satisfaction. While implant preservation is not universally feasible, this report suggests it may be a reasonable option in carefully selected patients when infection is detected early and managed aggressively (Table 1).

**Table 1:** Timeline of clinical events, management, and follow-up.

Day / Follow-up	Clinical Event	Management / Findings
0	Bilateral intramuscular buttock augmentation via intergluteal fold incisions	Prophylactic oral clindamycin initiated
5	Severe bilateral pain, left-sided swelling, erythema, systemic symptoms	Hospital admission; intravenous cefotaxime and metronidazole
6	CT: periprosthetic fluid collections, subcutaneous emphysema	Continued intravenous antibiotics
7	Spontaneous rupture of left incision with purulent drainage	Surgical reopening and saline irrigation; wound left open for drainage
10	Microbiology: coagulase-negative *Staphylococcus*	Antibiotics adjusted to ampicillin, clindamycin, ciprofloxacin
14	Persistent infection	Repeat irrigation; wound loosely approximated with adhesive strips
21	Infection resolution with intergluteal scarring	Discharged on prolonged oral antibiotics
180 (6 months)	Complete recovery, no recurrence of infection	Asymptomatic; full wound healing
365 (12 months)	Long-term follow-up	Stable implants, thin and well-healed bilateral scars, no late complications

## Conclusion

Intramuscular silicone buttock augmentation via the intergluteal fold offers aesthetic advantages but carries a clear risk of severe postoperative infection due to its anatomical location. This case demonstrates that, with early diagnosis, culture-guided antimicrobial therapy, and carefully selected wound management, implant preservation is achievable even in severe infections. Incorporating vigilant postoperative monitoring, individualized risk assessment, and thorough preoperative counseling can help optimise both patient safety and long-term aesthetic outcomes.

## Ethical considerations

Written informed consent was obtained from the patient for publication of this case report, including the use of anonymized clinical data and images. All identifying details have been removed to protect patient privacy. According to the policies of JPRAS Open, formal ethical committee approval was not required for a single-patient case report.

## Funding

This research received no specific grant from any funding agency in the public, commercial, or not-for-profit sectors.

## Declaration of Competing Interest

The authors declare no conflicts of interest relevant to this publication.
